# Confidence intervals for two sample means: Calculation, interpretation, and a few simple rules

**DOI:** 10.2478/v10053-008-0133-x

**Published:** 2013-06-17

**Authors:** Roland Pfister, Markus Janczyk

**Affiliations:** Department of Psychology III, Julius Maximilians University of Würzburg, Germany

**Keywords:** confidence intervals, graphical data presentation, repeated measures, within-subjects designs, between-subjects designs

## Abstract

Valued by statisticians, enforced by editors, and confused by many authors, standard errors (*SE*s) and confidence intervals (*CI*s) remain a controversial issue in the psychological literature. This is especially true for the proper use of *CI*s for within-subjects designs, even though several recent publications elaborated on possible solutions for this case. The present paper presents a short and straightforward introduction to the basic principles of *CI* construction, in an attempt to encourage students and researchers in cognitive psychology to use *CI*s in their reports and presentations. Focusing on a simple but prevalent case of statistical inference, the comparison of two sample means, we describe possible *CI*s for between- and within-subjects designs. In addition, we give hands-on examples of how to compute these *CI*s and discuss their relation to classical *t*-tests.

## 

*If you take care of the means, the end will take care of itself*. Mahatma Ghandi

## State of Affairs

Recent developments in psychological research methods converge on the notion that each mean is best accompanied by an appropriate confidence interval (*CI*) and, consequently, *CI*s are discussed in many contemporary statistical textbooks (e.g., Bortz & Schuster, 2010; Howell, 2012). Interestingly, this mainly holds true for between-subjects designs for which *CI*s are relatively easy to compute (Cumming & Finch, 2005). In contrast, standard errors (**SE**s) and *CI*s for within-subjects designs are still mysterious for many researchers (cf. Belia, Fiedler, Williams, & Cumming, 2005) even though several excellent publications elaborated on appropriate *CI*s for this situation during the last decades (Loftus & Masson, 1994; see also Cousineau, 2005; Estes, 1997; Franz & Loftus, 2012).

Most of these approaches to *CI*s for within-subjects desgins, however, are rather difficult to understand because they rely on relatively advanced measures such as the error term of the repeated-measures Analysis of Variance (ANOVA). So, while potentially applicable to a wide range of studies, *CI*s for within-subjects designs are widely misunderstood (Belia et al., 2005) and rather complicated to calculate (Bakeman & McArthur, 1996). This dilemma is especially relevant for cognitive psychologists who tend to rely heavily on within-subjects designs (e.g., as compared to researchers in personality or social psychology; cf. Erlebacher, 1977; Keren, 1993).

Nonetheless, reporting appropriate *CI*s has become an essential component of American Psychological Association (APA) style (APA, 2010; Wilkinson & the Task Force on Statistical Inference, 1999), and nowadays many journal editors encourage authors to add *CI*s or *SE*s when reporting their data. Further, as teachers we often push students to aid their data presentation with *CI*s, be it in-class data presentation or for presentations at scientific meetings. Yet, an easy, tutorial-like explanation on how to choose and calculate these *CI*s is missing. To fill this gap, we focus on a simple and often applied statistical analysis - the comparison of two means from independent groups as well as from dependent groups/conditions - and describe appropriate*CI*s in an intuitive framework. In this framework, we suggest that using a much simpler approach to within-subjects *CI*s than suggested in often-referenced papers (e.g., Loftus & Masson, 1994) is preferable in most cases. In particular, this approach simply relies on the *CI* of the difference between sample means (see Franz & Loftus, 2012, for a more detailed discussion of this approach and its advantages over other approaches). Such *CI*s are more initimately related to their between-subjects counterparts, are easily obtained with any computer program, and allow for a straightforward interpretation.

In the following section, we outline how different *CI*s can be computed for the common situation of comparing two sample means (cf. Table 1). These guidelines are intended to simplify graphical data presentation in a unifying framework that is intimately related to the different *t*-tests in classical hypothesis testing.

**Table 1.** Fundamental Concepts for the Graphical Data Presentation of Two Means and the Associated Confidence Intervals

Parameter	A parameter is a fixed, but unknown population value. Sample statistics are used to estimate parameters.
Standard error (*SE*)	Measure for the standard deviation of a parameter estimator. In case of a sample mean, it is equal to the estimated standard deviation divided by the square root of the underlying sample size.
Confidence interval (*CI*)	An estimate for plausible population parameters. Several different *CI*s can be constructed for the comparison of two means, depending on the employed design and the desired interpretation. Still, each *CI* can be broken down to the simple formula: "Mean ± Standard Error ✕ Coefficient" (*CI* = *M* ± *SE* ✕ t_df;1-1-α/2_
Confidence interval for an individual mean (*CI*_M_)	This *CI* is constructed from the standard error of the mean (*SE*_M_) and can be used to compare this mean to any fixed parameter. It corresponds to a one-sample *t*-test and does not yield any precise information about the difference between two sample means.
Confidence interval for the difference between two means from independent samples (*CI*_D_)	This *CI* is constructed from the between-subjects standard error of the difference between two means (*SE*_D_). It thus corresponds to a *t*-test for independent samples and can be used for inferences about the difference between both means.
Confidence interval for the paired difference between two means (*CI*_PD_)	This *CI* is constructed from the standard error of the difference between two dependent sample means (paired differences). It is thus applicable for within-subjects designs and equivalent to a paired-samples *t*-test.

## A *CI* is a *CI* is a *CI*

Independent of the underlying design, any *CI* for a sample mean can be broken down to a simple formula that only includes the mean itself, an appropriate *SE*, and a coefficient that is derived from the *t*-distribution. In the following, we will use 95% *CI*s (i.e., = .05) in all examples because of their widespread use in the literature (cf. Equation 1):

(1) $$95\%\mathrm{Cl}=M\pm \mathrm{SE}\cdot t_{\mathrm{df};1-\frac{0.05}{2}}$$

All *CI*s computed with this formula rely on the same assumptions as *t*-tests in classical hypothesis testing do. More precisely, they assume a normally distributed variable with an unknown population variance that is estimated from the sample (thus implying measurement at the interval scale). Furthermore, these *CI*s are inherently two-tailed, as reflected by the use of α/2 to determine the coefficient.

Most importantly, particular *CI*s differ in how the corresponding *SE* is computed, and the appropriate formula depends on two factors: (1) the experimental design and (2) the intended meaning of the *CI*. We start by discussing two *CI*s for between-subject designs before continuing with within-subjects designs. Following these theoretical points, we demonstrate how to compute the three *CI*s for an exemplary data set.

### Between-subjects designs: Two independent samples

For between-subjects designs, two distinct *CI*s can be computed that differ in meaning and interpretation. At first sight, the most straightforward way might be to compute separate *CI*s for each individual mean *M* by simply using the corresponding *SE*. In fact, this is a valid solution and we will denote the resulting *SE* as *SE*_M_. Following from the central limit theorem, *SE*_M_ is computed by dividing the unbiased estimator of the standard deviation (*s*) by the square root of the sample size *n* (see Equation 2):

(2) $${\mathrm{SE}}_{M}=\frac{s}{\sqrt{n}}\mathrm{with}s=\sqrt{s^{2}}=\sqrt{\frac{1}{\left(n-1\right)}\cdot \sum_{i=1}^{n}{\left(x_{i}-M_{x}\right)}^{2}}$$

The corresponding *CI* for individual means is denoted as *CI*_M_ (cf. Equation 3):

(3) $$95\%{\mathrm{Cl}}_{M}=M\pm {\mathrm{SE}}_{M}\cdot t_{n-1;0.975}$$

This confidence interval can be used for inferring whether the mean is different from any fixed, hypothesized value (e.g., zero). Thus, the *CI*_M_ corresponds to the one-sample *t*-test. The *CI*_M_, however, does not yield any precise information about the difference between its mean and any other sample mean. To obtain this information, one needs to compute a different *SE* that captures the (between-subjects) variability of the difference between both means. This measure - *SE*_D_ - is composed of both sample sizes (*n*_1_, *n*_2_) and estimated standard deviations (*s*_1_ and *s*_2_, respectively; see Equation 4):

(4) $${\mathrm{SE}}_{D}=\sqrt{\frac{\left(n_{1}-1\right)s_{1}^{2}+\left(n_{2}-1\right)s_{2}^{2}}{\left(n_{1}+n_{2}-2\right)}}\cdot \sqrt{\frac{1}{n_{1}}+\frac{1}{n_{2}}}$$

The corresponding *CI* for the difference between two independent means is denoted as *CI*_D_ (cf. Equation 5):

(5) $$95\%{\mathrm{Cl}}_{D}=M_{1/2}\pm {\mathrm{SE}}_{D}\cdot t_{n_{1}+n_{2}-2;0.975}$$

In addition to the general assumptions mentioned above, *CI*_D_ assumes that the standard deviations are estimated from independent samples and that the size of these standard deviations is comparable (i.e., we assume homogeneity of variance)^1^. Importantly, conclusions based on the *CI*_D_ are valid only for the difference between the means, and the *CI*_D_ thus corresponds to the *t*-test for two independent samples. If centered around one of the means this test is significant if, and only if, the *CI*_D_ does not include the other mean. Accordingly, the *CI*_D_ can be used for inferences about the statistical significance of the between-subjects difference; and because the difference between sample means is what a researcher will typically be interested in, *CI*_D_ is preferable to *CI*_M_ in most circumstances.

To conclude, *CI*_D_ and *CI*_M_ are intimately related to classical *t*-tests and allow for a straightforward interpretation: A standard *t*-test is significant if, and only if, the 95% *CI* does not include the value in question. For the *CI*_D_, this value is the second sample mean, for the *CI*_M_, this is any fixed parameter value.

### Within-subjects designs: Two paired samples

For within-subjects designs, matters seem to be more complicated at first sight. In fact, Cumming and Finch (2005) recommended: “For paired data, interpret the mean of the differences and error bars for this mean. In general, beware of bars on separate means for a repeated-measure independent variable: They are irrelevant for inferences about differences“ (p. 180).

Caution is indeed necessary in this situation, because the often-used *CI*_M_ obviously is unrelated to the within-subjects difference. Yet, several *CI*s for within-subjects designs have been proposed in the last decades (Cousineau, 2005; Jarmasz & Hollands, 2009; Loftus & Masson, 1994) with the most prevalent variant being the one of Loftus and Masson. These *CI*s are typically derived from the error term of the repeated-measures ANOVA and we will come back to these methods in the section *What to do with more complex designs*. For comparing the means of two paired samples, however, a straightforward and elegant solution seems to be more closely related to meaning and interpretation of *CI*s for between-subjects designs (see also Franz & Loftus, 2012). This solution simply uses the standard error of the paired differences (*SE*_PD_) to construct the *CI*. Accordingly, it does not require any ANOVA statistics, but can be computed easily from the standard deviation of individual difference scores *s*_d_ (see Equation 6):

(6) $${\mathrm{SE}}_{\mathrm{PD}}=\frac{s_{d}}{\sqrt{n}}$$

The corresponding *CI* is labelled *CI*_PD_ (following Franz & Loftus, 2012; cf. Equation 7

(7) $$95\%{\mathrm{Cl}}_{\mathrm{PD}}=M_{1/2}\pm {\mathrm{SE}}_{\mathrm{PD}}\cdot t_{n-1;0.975}$$

The *CI*_PD_ is thus equivalent to the confidence interval of the difference between both paired means and corresponds directly to a paired-samples *t*-test. When plotted around the actual sample means, this *t*-test is significant if one mean is not part of the *CI*_PD_ around the other mean; consequently *CI*_PD_ is a direct within-subjects counterpart of the *CI*_D_ for independent samples. Taken together, we suggest that the *CI*_PD_ can be computed more easily and seems to be more closely related to interpreting the difference between two dependent means than any other solution.

## Affection, pheromones, and *CI*s: A hands-on example

Table 2 shows the data of a fictitious - and rather arbitrary - study in which participants indicated their affection for the experimenter on a rating scale. This scale ranges from -10 (*dislike*) to 0 (*neutral*) to 10 (*affection*). Condition 1 is a control condition without any specific treatment whereas the experimenter used a healthy dose of pheromones in Condition 2.

**Table 2.** Example Data

Reported affection for the experimenter as indicated on a rating scale (-10 to 10).		
Observation	Condition 1(control)	Condition 2(pheromones)
1	7	8
2	3	5
3	4	6
4	2	5
5	5	7
M	4.20	6.20
*s*	1.92	1.30

*Note*. Condition 1 is a control condition without any specific treatment, whereas the experimenter had used a dose of pheromones in Condition 2. In the following equations, we will use the indices *1* and *2* to refer to the control condition and the pheromone condition, respectively.

**Figure 1.**
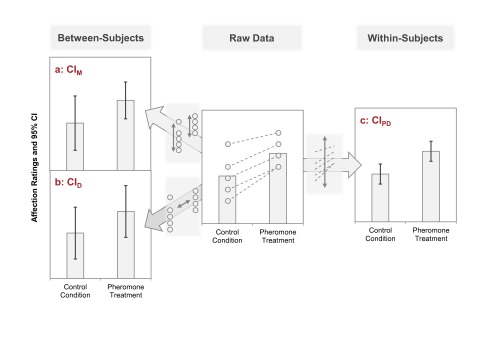
Three different confidence intervals (*CI*s) for two sample means. The raw data are plotted in the center of the figure; dots represent individual data points (five observations per mean; see also Table 2). Panels A and B show *CI*s that are appropriate for *between-subjects designs*; Panel C shows a *CI* that is appropriate for *within-subjects designs* (pairs of values are indicated by dashed lines in the raw data). Panel A. *CI*s for individual means (*CI*_M_) rely on the standard error (*SE*) of the corresponding mean. The *CI*_M_ indicates whether this mean is significantly different from any given (fixed) value. They do not inform about the statistical significance of the difference between the means. Panel B. *CI* for the difference between the means (*CI*_D_). The means are significantly different (as judged by t-tests for independent samples) if one mean is not included in the *CI*_D_ around the other mean. Panel C. Within-subjects *CI*, constructed from the paired difference scores (*CI*_PD_). Two means from paired samples are significantly different (as judged by a paired-samples *t*-test) if one mean is not included in the *CI*_PD_ around the other mean.

Different *CI*s are possible in this situation, depending on the actual design and the *CI*’s intended meaning. The most important question, of course, relates to the design: Different *CI*s are appropriate depending on whether the data result from a between-subjects design (different participants contributed to Condition 1 and Condition 2, respectively) or a within-subjects design (the data in each row belong to a single participant). The three different *CI*s described above are plotted in Figure 1 and will be discussed in the following (see Appendix A for a short tutorial on how to compute these intervals with common computer programs).

### Between-subjects: *CI*s for individual means

Confidence intervals for individual means can be easily computed based on the two standard deviations in Table 2. Accordingly, the two *SE*s amount to the following values (Equation 8):

(8) $${\mathrm{SE}}_{M\left(1\right)}=\frac{s_{1}}{\sqrt{n_{1}}}=\frac{1.92}{\sqrt{5}}=0.86{\mathrm{SE}}_{M\left(2\right)}=\frac{s_{2}}{\sqrt{n_{2}}}=\frac{1.30}{\sqrt{5}}=0.58$$

Both *SE*s are then multiplied with the critical *t*-value of t5-1; 0.975 = 2.78 to compute the respective *CI* (see Equation 9 and 10). These two *CI*_M_ are plotted in Figure 1 (Figure 1, Panel A).

(9) $$95\%{\mathrm{Cl}}_{M\left(1\right)}=4.20\pm 0.86\cdot 2.78=4.20\pm 2.39$$

(10) $$95\%{\mathrm{Cl}}_{M\left(2\right)}=6.20\pm 0.58\cdot 2.78=6.20\pm 1.62$$

The two *CI*_M_ indicate that the mean affection ratings are significantly different from zero for both conditions, that is, participants were positively biased toward the experimenter even when not affected by pheromones. Importantly, however, the *CI*_M_ are not informative for the difference between the affection rating of the control participants and the participants who were exposed to pheromones.

### Between-subjects: *CI*s for the difference

The *SE*_D_ is equivalent to the *SE* that is used for the *t*-test for independent samples (Equation 11):

(11) $${\mathrm{SE}}_{D}=\sqrt{\frac{\left(n_{1}-1\right)s_{1}^{2}+\left(n_{2}-1\right)s_{2}^{2}}{\left(n_{1}+n_{2}-2\right)}}\cdot \sqrt{\frac{1}{n_{1}}+\frac{1}{n_{2}}}=\sqrt{\frac{\left(5-1\right)\cdot 1.92^{2}+\left(5-1\right)\cdot 1.30^{2}}{\left(5+5-2\right)}}\cdot \sqrt{\frac{1}{5}+\frac{1}{5}}=1.04$$

Because the difference between both (independent) means involves subjects of both groups, the *CI*_D_ is computed by multiplying the *SE*_D_ with the appropriate critical *t*-value of t5 + 5 - 2; 0.975 = 2.31 (see Equation 12):

(12) $$95\%{\mathrm{Cl}}_{D}=M_{1/2}\pm 1.04\cdot 2.31=M_{1/2}\pm 2.40$$

This *CI*_D_ is plotted around each mean in Figure 1 (Figure 1, Panel B). The mean rating of the control participants is clearly included in the *CI*_D_ around the mean of the participants who were exposed to pheromones - both values are thus not significantly different as judged by a *t*-test for independent samples.

### Within-subjects: *CI* for the difference

In contrast to the previous *CI*s, we now assume the data in Table 2 to result from a within-subjects design: Participants were first tested in the control condition and then exposed to the pheromones (or vice versa). Accordingly, the two ratings in each row of Table 2 are now assumed to belong to the same individual. The now appropriate *CI*_PD_ is based on the pairwise difference scores for the data in Table 2 (Condition 2 - Condition 1). These differences scores are 1, 2, 2, 3, and 2 for the five participants and their standard deviation is *s*_d_ = 0.71. The corresponding *SE*_PD_ amounts to (see Equation 13):

(13) $${\mathrm{SE}}_{\mathrm{PD}}=\frac{s_{d}}{\sqrt{n}}=\frac{0.71}{\sqrt{5}}=0.32$$

With a critical *t*-value of t_5 - 1; 0.975 = 2.78_, we can now compute the *CI*_PD_ (Equation 14):

(14) $$95\%{\mathrm{Cl}}_{\mathrm{PD}}=M_{1/2}\pm 0.32\cdot 2.78=M_{1/2}\pm 0.88$$

The *CI*_PD_ is plotted around each mean in Figure 1 (Figure 1, Panel C). The mean of the control condition is clearly not included in the *CI*_PD_ around the mean of the pheromones condition. This is equivalent to a significant effect as revealed by a paired-samples *t*-test.

## Deciding what to plot

As we have seen in the above example, different and equally possible *SE*s and *CI*s for a given situation can vary substantially and do convey different information. On closer inspection, the question which one to plot boils down to the question whether the difference between the two means is of major interest or not.

If the difference is indeed of interest, we suggest that each mean is best accompanied by the *CI* of the difference that is appropriate for the employed design (i.e., either *CI*_D_ or *CI*_PD_). As noted above, these intervals allow direct inferences about the difference and have also been labelled *inferential* *CI*s for this reason (Tryon, 2001). In addition to plotting these *CI*s, it is of course equally important to describe what is plotted. Here, a typical description to be used in a figure caption would be “Error bars represent the XY% confidence interval of the difference”. Alternatively, a concise description is also possible with the nomenclature suggested in this article that can be used to specify the plotted *CI* or *SE* on the axis of a graph (e.g., “RT ± *SE*_PD_” or “RT and *CI*_PD_” for a within-subjects design using response time as dependent variable).

An additional option to this approach can be used if it is only the difference that counts whereas the actual means are not of interest. In this case, it is also possible to plot only the difference itself, accompanied by the corresponding *CI* (i.e., *CI*_D_ or *CI*_PD_).

If the difference between the two means is not of major interest, however, we suggest to plot the *CI*_M_ or *SE*M for each individual mean. Here, a typical description to be used in a figure caption would be “Error bars represent the XY% confidence interval of the individual means” or, to use the suggested nomenclature, a similar statement on the axis of a graph (e.g., “RT ± *SE*M” or “RT and *CI*_M_”). These error bars inform about the homogeneity of variance across different samples or conditions and - even though they cannot be used for inferences about the difference between two means - they provide information about the difference of each mean from a fixed parameter.

## What to do with more complex designs?

The framework described in the preceding sections provides a straightforward and intuitive approach to *CI*s for means from two conditions for both, between- and within-subjects designs. These *CI*s can be mapped directly to the different *t*-tests in classical hypothesis testing and, as mentioned above, they also rely on the same statistical assumptions as the corresponding test. The described method of plotting the appropriate *CI* for the difference - *CI*_D_ or *CI*_PD_, respectively - can also be applied to more complex designs given that specific pairwise comparisons are crucial for the research question at hand (Franz & Loftus, 2012). If applicable, this method might indeed be the easiest and thus favorable strategy.

Still, this approach has obvious limitations regarding complex stu-dies which include numerous conditions. In such factorial designs, *CI*s are typically constructed from the error term of the ANOVA omnibus test. For between-subjects designs, appropriate methods are described comprehensively in several publications (e.g., Keppel & Wickens, 2004; cf. also Estes, 1997). As noted above, different methods have been proposed also for factorial within-subjects designs (Cousineau, 2005; Jarmasz & Hollands, 2009; Loftus & Masson, 1994) with the most prevalent variant being the one of Loftus and Masson (1994; see also Baguley, 2012; Bakeman & McArthur, 1996; Masson & Loftus, 2003; Hollands & Jarmasz, 2010; Tryon, 2001). Using these methods, however, requires a precise understanding of what these *CI*s reveal about the data. For instance, within-subject *CI*s according to Loftus and Masson (1994) are not directly equivalent to *t*-tests for paired samples but have to be multiplied by a fixed factor to allow for inferences about possibly significant effects (i.e., in the case of two groups/conditions: *CI*_PD_ = 2 × *CI*_Loftus& Masson_). Excellent examples on how to compute and interpret these *CI*s can be found in the corresponding articles.

## Concluding Remarks

In the preceding sections, we have summarized three approaches to *CI*s for one of the most common designs in psychological research, that is, the comparison of two sample means. Clearly, different *CI*s need to be computed for between- and within-subjects designs (cf. Blouin & Riopelle, 2005; Cumming & Finch, 2005; Estes, 1997; Loftus & Masson, 1994) and the particular *CI* used in a plot needs to be specified. To this end, we suggested an easy nomenclature for three different *CI*s to facilitate communication about what exactly a given *CI* represents (see Table 1). Furthermore, we argue that *CI*s for the difference between two means (*CI*_D_ and *CI*_PD_) are most informative in the majority of cases, because they can be interpreted intuitively. These *CI*s provide a straightforward approach to the described setting; more complex designs of course call for different approaches to *CI*s which can be found in a variety of recent articles.

## Figures and Tables

**Figure A1.**
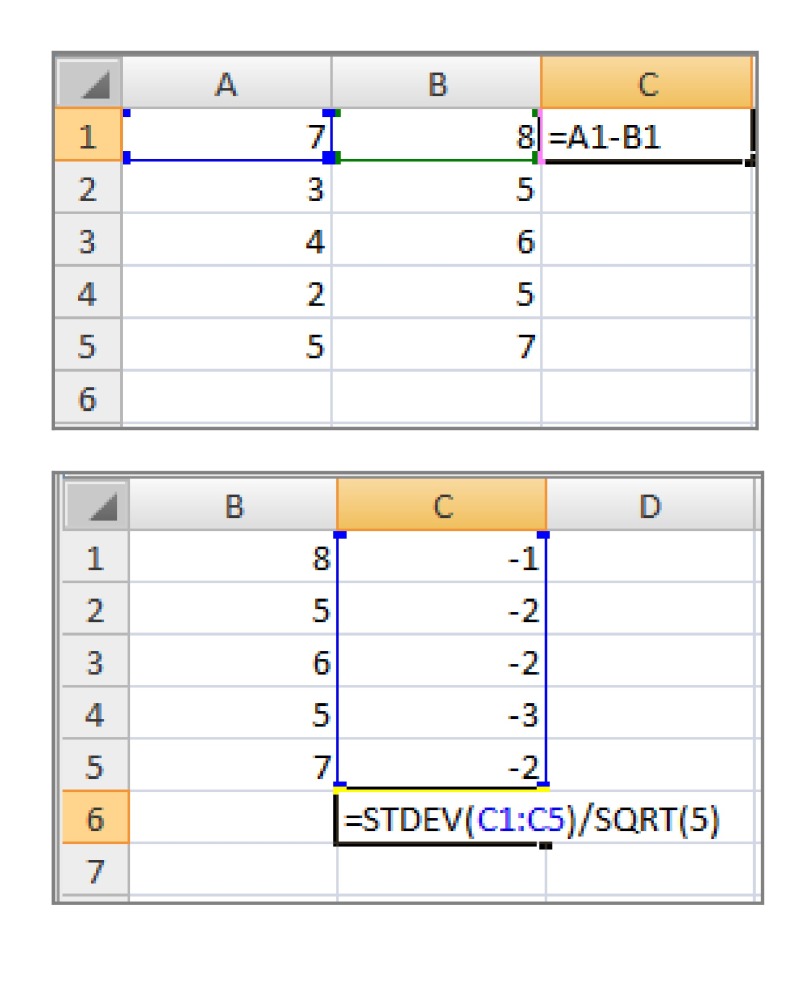
Computing the *SE*_PD_ in MSMS Excel. Upper panel: Pairwise differences are computed for each case. Lower panel: The standard deviation of these difference scores is divided by √*n* to obtain the *SE*_PD_.

## Footnotes

1 In the rare case of two equally sized samples with *numerically* identical standard deviations, the *SE*_M_ is informative also for the difference between the means. Here, it is directly related to *SE*_D_ with *SE*_D_ = 2 × *SE*_M_. If sample sizes or standard deviations are (even slightly) dissimilar, however, this relation is not valid. It should also be noted that this relation is only valid for *SE*s but not for the corresponding *CI*s: The coefficient of the *CI*_M_ has *n* - 1 degrees of freedom (*df*) whereas the coefficient of the *CI*_D_ has (*n*_1_ + *n*_2_ - 2)*df*.

## Appendix A

In the following, we show how the different *CI*s can be computed by common software packages, such as SPSS, MS Excel, and R.

### SPSS

*CI*s for individual means (*CI*_M_) can be computed with the *Explore* command:

*Analyze* > *Descriptive Statistics* > *Explore*

Here, the menu Statistics allows to set the α level (default: 5%). Alternatively, the *CI*_M_ is also contained in the output of the one-sample *t*-test in a section labelled 95% *Confidence Interval of the Difference* (with the α level being set in the *Options* menu). Both ways of computing the *CI* result in a *CI* that is specified via lower and upper boundaries which can be easily transformed to the the notation that is used in this article (see Equation A1):

(A1) $${\mathrm{Cl}}_{M}=M\pm \frac{\mathrm{Upper}-\mathrm{Lower}}{2}$$

*CI*s for the difference between independent means (*CI*_D_) and *CI*s for the difference between paired means (*CI*_PD_) can be obtained by using the same formula on the output of the *t*-test for independent-samples and the paired-samples *t*-test, respectively. These outputs also contain the corresponding values for *SE*_D_ or *SE*_PD_.

### Microsoft Excel

Computing *CI*s for individual means (*CI*_M_) in MS Excel requires several quick steps. First, the standard deviation *s* is computed with *STDEV* function. Dividing this value by √*n* returns the *SE*_M_. This can be done by using *SQRT*(*n*) or by computing *n* with the *COUNT* function. Finally, the *SE*_M_ is multiplied with the coefficient taken from the *t*-distribution which can be accessed via the *TINV* function. Importantly, *TINV* is inherently two-tailed and takes the intended α level as input. Thus, calling *TINV*(0.05, *n*-1) will result in the correct value for *t*_*n* - 1; 0.975_. The *CI* for the difference of two independent means, *CI*_D_, is computed similarly with two changes. Most importantly, the *SE*_D_ is computed using the corresponding formula in the section *Between-subjects designs: Two independent samples* (using *SQRT* for the square root and “^2” to denote exponents). Furthermore, the critical *t*-value has to be requested with the correct number of *df*s via *TINV*(0.05, *n*_1_ + *n*_2_ - 2). In contrast to *CI*_M_ and *CI*_D_, the *CI* for the difference between paired means, *CI*_PD_, requires an additional first step. Assuming that each condition is entered in a separate column, one first needs to compute pairwise differences (Figure A1). The estimated standard deviation of the difference scores *d*_d_ can now be computed via the *STDEV* function. Dividing this value by √*n* returns the *SE*_PD_. This can again be done by using *SQRT*(*n*). *CI*_PD_ is then computed by multiplying the *SE*_PD_ with the coefficient computed by *TINV*(0.05, *n*-1) for *t*_*n* - 1; 0.975_.

### R

Confidence intervals are included in the output of the function *t.test*. As in SPSS, *CI*s are typically given in terms of lower and upper boundaries. These values can be accessed directly to arrive at the notation that is used in this article:

(A2) $${\mathrm{Cl}}_{M}=M\pm \frac{\mathrm{Upper}-\mathrm{Lower}}{2}$$

Accessing the boundaries works similarly for all *t*-tests and we will demonstrate the general procedure for the one-sample *t*-test and the corresponding *CI*_M_. First, we enter the data of Condition 1 as a vector and compute the one-sample *t*-test via *t.test*. The output is stored in the new variable *result*:

>*cond1 <- c(7,3,4,2,5)*

>*result <- t.test(cond1)*

The boundaries can now be addressed by *result$conf.int* which returns a vector containing both values. The length of an individual error bar can now be computed in the following way:

>*(max(result$conf.int) - min(result$conf.int))/2 [1] 2.388388*

Alternatively, R allows for a manual computation of *SE*s and *CI*s just as in MS Excel. We demonstrate this procedure for the *CI*_PD_. For the sake of simplicity, we assume the data to be coded in vectors instead of being variables in a data frame.

>*cond1 <- c(7,3,4,2,5)* >*cond2 <- c(8,5,6,5,7)*

We then compute a difference vector and its length (i.e., the number of participants):

>*diff < -cond1-cond2 >n <-length(diff)*

Then, the standard deviation of these difference scores is divided by √*n* and multiplied by *n*_n - 1; 0.975_. The latter coefficient is computed via the function *qt*.

>*errorbar < -sd(diff)/sqrt(n)*qt(0.975, n-1)*

The resulting error bar is used to compute upper and lower boundaries of the *CI*_PD_ for both condition means:

>*ci1 < -c(mean(cond1)-errobar,mean(cond1)+errobar*

>*ci2 < -c(mean(cond2)-errobar,mean(cond2)+errobar*
